# Toward a Full Configurational Accuracy Calculation of an Arbitrary Molecule via Fragment Embedding and a Stochastic Solver

**DOI:** 10.1021/acs.jpclett.4c00634

**Published:** 2024-04-11

**Authors:** Yi Sun

**Affiliations:** Department of Chemistry, Chicago Center for Theoretical Chemistry, James Franck Institute, and Institute for Biophysical Dynamics, The University of Chicago, Chicago, Illinois 60637, United States

## Abstract

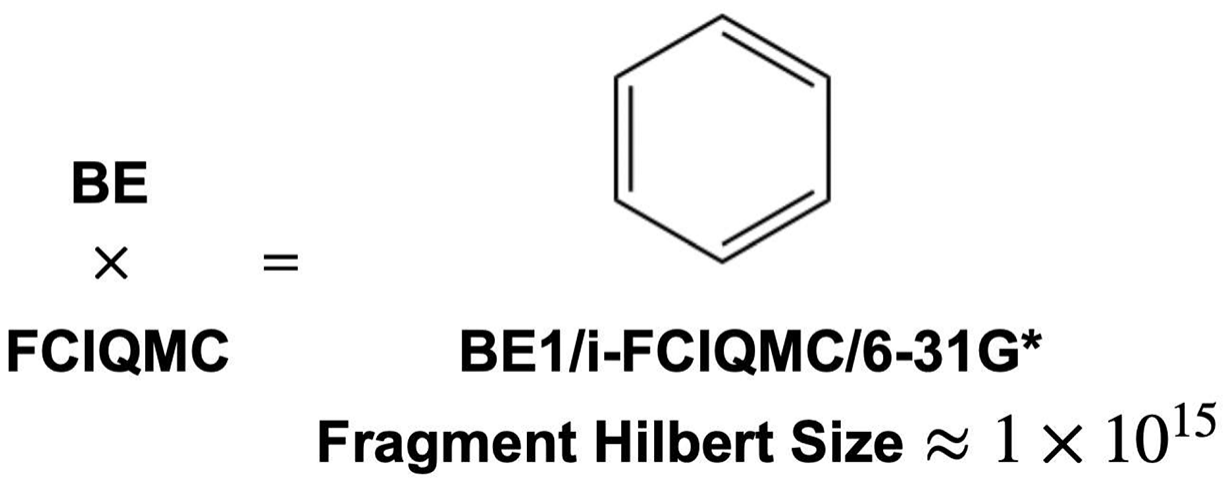

We demonstrate the feasibility of using a stochastic solver, full configuration interaction quantum Monte Carlo with the initiator approximation (i-FCIQMC), to converge fragment embedding calculations, namely bootstrap embedding (BE). We first propose and test a general protocol for converging BE-i-FCIQMC calculations and then suggest how the quality of the calculation compares against that of deterministic BE-FCI using different numbers of walkers. We then demonstrate that BE-i-FCIQMC can perform as well as BE-FCI in the large walker limit and how different factors, including the size of the Hilbert space of the fragments, the number of walkers, and the nature of the chemical system, affect the achievable matching error. We finally perform BE-FCI calculations in realistic systems like benzene and cyclohexane using a double-ζ basis set. This work demonstrates the potential of performing FCI quality calculations in realistic systems using BE.

There are a number of methods for tackling the electronic structure problem, such as Hartree–Fock theory (HF),^1,2^ density functional theory (DFT),^3,4^ coupled cluster theory,^5−9^ and full configuration interaction (FCI). Among them, FCI has long been known as the method that can provide numerically exact solutions to the time-independent Schrödinger equation under a specific basis set.^3,10−12^ Nevertheless, its exponential scaling with the number of electrons and orbitals prevents it from being used to calculate the energies of larger systems. In this work, we combine a stochastic method, full configuration interaction quantum Monte Carlo (FCIQMC),^13−16^ and the fragment embedding method, bootstrap embedding (BE),^17−21^ to calculate molecular energies at the FCI level and examine the scaling relationship between the size of the Hilbert space of the fragments and the number of walkers required to maintain the quality of the solution.

We first outline the key principles behind FCIQMC and BE. FCIQMC is a stochastic electronic structure method that is inspired by projector Monte Carlo.^22^ If a Wick rotation is performed on the time-dependent Schrödinger equation (TDSE) and *ℏ* is defined to be 1, the TDSE can be reformulated^22^ as
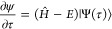
1where τ = *it*. Defining the state |Ψ(τ = 0)⟩ = |Φ_0_⟩ and decomposing |Φ_0_⟩ in the basis of the Hamiltonian eigenfunctions, *Ĥ*|Ψ_*i*_⟩ = *E*_*i*_|Ψ_*i*_⟩, gives |Φ_0_⟩ = ∑_*i*_*c*_*i*_|Ψ_*i*_⟩. Assuming that eigenfunctions are ordered according to the energy |Ψ_0_⟩ is the ground state with energy *E*_0_ and requiring that *c*_0_ be non-zero, one can show that when τ → *∞*
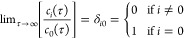
2thus recovering the ground state wave function; the corresponding energy value can be computed variationally from  or by projection from . Therefore, if the FCI wave function ansatz, |Ψ_FCI_⟩ = ∑_*i*_*C*_*i*_|*D*_*i*_⟩, is propagated through time, one can obtain the corresponding updating equation for each coefficient, *C*_*i*_, via finite difference
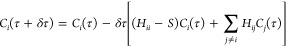
3where *H*_*ij*_ = ⟨*D*_*i*_|*Ĥ*|*D*_*j*_⟩ and *S* has replaced *E* to take the role of a population control parameter. Coefficients, *C*_*i*_, are represented by populations of “walkers” on Slater determinants, and the goal of population control is to make sure that the number of walkers is controlled to be below a certain value. FCIQMC then uses the corresponding spawning and death steps to perform this propagation stochastically, resulting in the FCI solution. Several important modifications can improve the performance of FCIQMC, including the initiator method (i-FCIQMC), introduced in 2010 by Cleland et al.,^14^ that made it possible to converge the calculation with a lower number of walkers, and a further modification known as the adaptive shift method^23^ can reduce the initiator error and allows one to obtain near-FCI quality results for systems that have a Hilbert space size of 10^35^ using 2 × 10^8^ walkers, which is 10^25^ times larger than the largest FCI calculation reported to date.^24^ Additionally, semistochastic adaptations of FCIQMC make it possible to deterministically propagate a number of determinants (usually the most populated ones), which dramatically reduces the stochastic noise.^25,26^

BE attempts to break down the calculation of a whole molecule into overlapping fragments. For a chemical system described by a second-quantized Hamiltonian
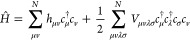
4where **h** and **V** are the standard one- and two-electron integrals between the N orbitals, respectively, and *c*_μ_^†^ (*c*_μ_) creates (annihilates) an electron in a local orbital (LO), |ϕ_μ_⟩. The LOs can be obtained from the Foster–Boys localization method,^27^ using intrinsic atomic orbitals (IAOs),^28^ etc., and form an orthonormal basis. Suppose the HF solution of the Hamiltonian *Ĥ* is |Φ_0_⟩. We define a fragment *A* by specifying a subset of *N*_*A*_ LOs on *A*, {ϕ_μ_}_μ∈*A*_. Typically, *N*_*A*_ ≪ *N* as the number of electrons, *N*_*A*_, on an embedding fragment is always smaller than that in a molecule, which is *N*. Then it can be shown that the HF state of a molecule can be decomposed as the following product:
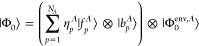
5

Equation 5 is called a Schmidt decomposition (SD) of |Φ_0_⟩ on fragment *A* and divides the system into three parts: the fragment orbitals (FOs) {*f*_*p*_^*A*^}_*p*=1_^*N*_*A*_^ that we choose to be the fragment LOs {ϕ_μ_}_μ∈*N*_*A*__, the bath orbitals (BOs) {*b*_*p*_^*A*^}_*p*=1_^*N*_*A*_^ that are entangled with the FOs, and the frozen core |Φ_0_^env,*A*^⟩ that is disentangled with the FOs. The 2*N*_*A*_ FOs + BOs are called the embedding orbitals (EOs). It is worth mentioning that all of the BOs are assumed to be entangled with FOs, which means that 0 < η_*p*_^*A*^ < 1 for all *p*.

The embedding Hamiltonian can be derived by realizing that the EOs span an active space, with the environment being a spectator. The form of it is shown below:
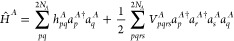
6where
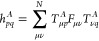

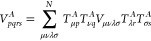
7where **T**^*A*^ = [**T**^*f*,*A*^|**T**^*b*,*A*^] is the coefficient matrix of {|*f*_*p*_^*A*^⟩} and {|*b*_*p*_^*A*^⟩} expanded in the set of LOs. As the size of *Ĥ*^*A*^ is usually much smaller than the original Hamiltonian due to the inclusion of fewer orbitals (if 2*N*_*A*_ < *N*), computational resources will be reduced in fragment embedding calculations when compared to a full system calculation using the same wave function solver.

Density matrix embedding theory (DMET) is another method that utilizes fragment embedding to estimate the wave function and the energy of a molecule. The main difference between DMET and BE is the matching condition. Because DMET normally uses non-overlapping fragments, it aligns the density matrix of the mean field with that of the correlated wave function computation for each fragment. On the contrary, BE utilizes overlapping fragments and aligns the density matrix of one fragment with that of another within their shared region of overlap. Therefore, matching in BE can be expressed mathematically as a collectively restricted optimization problem. To briefly describe how this is done, consider two overlapping fragments, *A* and *B*, which is shown in Figure 1.

**Figure 1:**
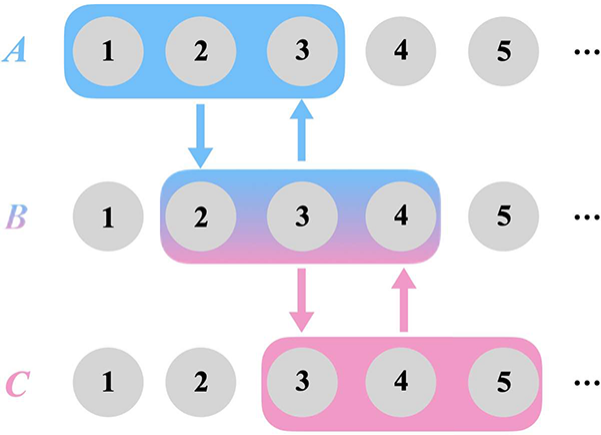
Schematic illustration of the BE matching conditions on a one-dimensional lattice model.

Let _*A*_ be the set of edge LOs of *A* (LO 1 and 3 in Figure 1) and _*B*_ be the center LOs of *B* (LO 3 in Figure 1). Then, _*A*_ ∩ _*B*_ denotes the overlapping region where the density matrix of *A* to that of *B* should be matched, which is LO 3 in Figure 1. If only matching of the elements of the 1-PDM of different fragments is considered, the fragment calculation of *A* is then constrained as follows:
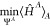
8subject to
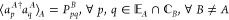
9where ⟨···⟩_*A*_ = ⟨Ψ^*A*^|···|Ψ^*A*^⟩. As Ψ^*A*^ is the wave function of fragment *A*, *P*_*pq*_^*B*^ can then be defined as the 1-PDM of the overlapping orbitals between fragments *A* and *B*. We loop over all fragments *B* ≠ *A* to enumerate all of the matching conditions for *A*. One can apply this to all fragments, leading to the following Lagrangian for constrained optimization when summing over all of the fragments:
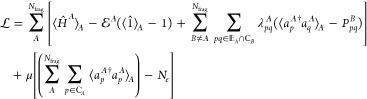
10in which {λ_*pq*_^*A*^} are the Lagrange multipliers for the density matching and μ is a global chemical potential that fixes the total number of electrons for non-overlapping fragment centers. The stationary points of eq 10 are described by the eigenvalue equation for fragment *A*:
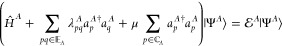
11which, in addition to the embedding Hamiltonian *Ĥ*^*A*^ (see eq 4), includes local effective potential term λ̂^*A*^, as well as a global chemical potential μ term. An iterative scheme can be designed to adjust μ and λ̂^*A*^ to converge the calculation by reducing the root-mean-square error, or the matching error ε, which is defined as
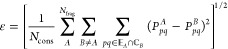
12decreases below some threshold value τ_BE_ (where *N*_cons_ is the total number of constraints). One can then obtain the BE energy value using the 1- and 2-PDMs of the fragments. The accuracy of the calculation will ultimately depend on the choice of the embedding fragments and the high-level solver.

When BE and FCIQMC are combined, they can calculate not only larger fragments at the FCI level but also larger systems by breaking them down into smaller fragments. This lets BE-FCIQMC calculate FCI quality energies in even larger molecules while using much less memory than an FCI calculation in the whole molecule, for this paper a program that interfaced between NECI^29^ to perform FCIQMC and the in-house BE code to carry out the calculations.

As a proof of concept, calculations were performed in linear H_8_ using the STO-3G basis set, with the type of BE being BE2. The meaning of *n* in BEn in terms of fragment partition means that in addition to the central atom, other atoms that are in the (*n* – 1)th coordination shell are also included in a fragment. Therefore, with respect to partitioning a chain-like molecule via BE2, each fragment always contains three atoms, including the central atom and the two nearest neighbors around this atom. A more detailed description of the partitioning scheme for arbitrary systems can be found in the relevant literature.^20^Figure 2 shows the structure of linear H_8_ and how the molecule is partitioned via BE2. Because bath orbitals are also involved in the embedding process, there are six orbitals and six electrons (instead of three) in each BE equation that needs to be solved, and the Hilbert space of the embedding fragment is 400 Slater determinants. Intrinsic atomic orbitals (IAO) are used as the LOs in the fragments.^28^ The Schmidt decomposition of the molecular wave function and the partitioning of the fragments were performed using in-house code, while NECI was interfaced and used to solve the BE equations stochastically. More simulation details, including how the integrals are generated and how the 1- and 2-PDMs are sampled, are available in the Supporting Information and the relevant literature.^15^ In addition, the initiator approximation is used to accelerate the calculation, making the solver i-FCIQMC, and for every BE-i-FCIQMC calculation, all of the determinants up to doubles are allowed to propagate deterministically to reduce the stochastic error.

**Figure 2:**
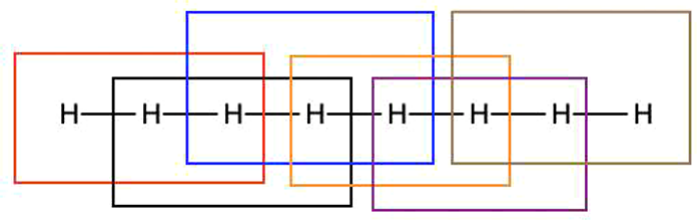
Structure of linear H_8_ as well as a depiction of the fragmentation of the molecule, where the interatomic separation between adjacent hydrogen atoms is 1 Å.

To test how the number of walkers affects the matching error and the quality of the calculation, five different numbers of walkers were chosen, namely, 50, 100, 250, 500, and 1000. Four test calculations were first carried out in parallel to see how to minimize the matching. The results are shown in Figure 3.

**Figure 3:**
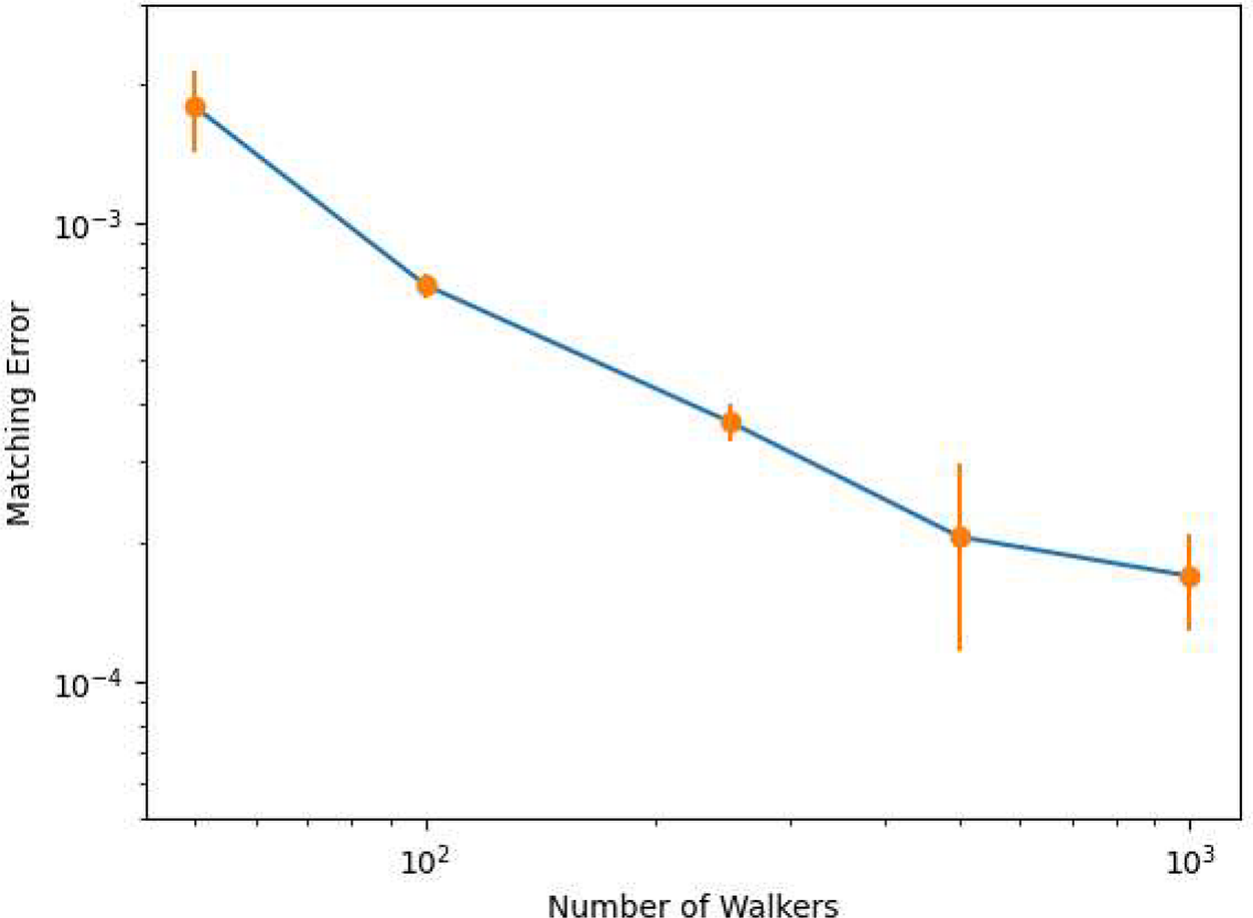
Relationship between the number of walkers and the minimum matching error with the error bars shown in H_8_.

One can see that there is a sharp decrease in the minimum matching error, which can be explained by the increase in the granularity of the i-FCIQMC wave function as the number of walkers increases. The thresholds of the matching error to converge a calculation are therefore set to 2.0 × 10^–3^, 8.0 × 10^–4^, 5.0 × 10^–4^, 2.5 × 10^–4^, and 2.0 × 10^–4^ for five different numbers of walkers. Figure 4 shows the proportion of the average correlation energy recovered from six parallel calculations relative to a deterministic BE-FCI, in which the convergence threshold is set to 1 × 10^–6^. It is worth noticing that before the stochastic noise significantly affects the matching error, i.e., at a similar order of magnitude, the number of iterations needed to converge the BE-FCI and BE-i-FCIQMC to a designated matching error is similar.

**Figure 4:**
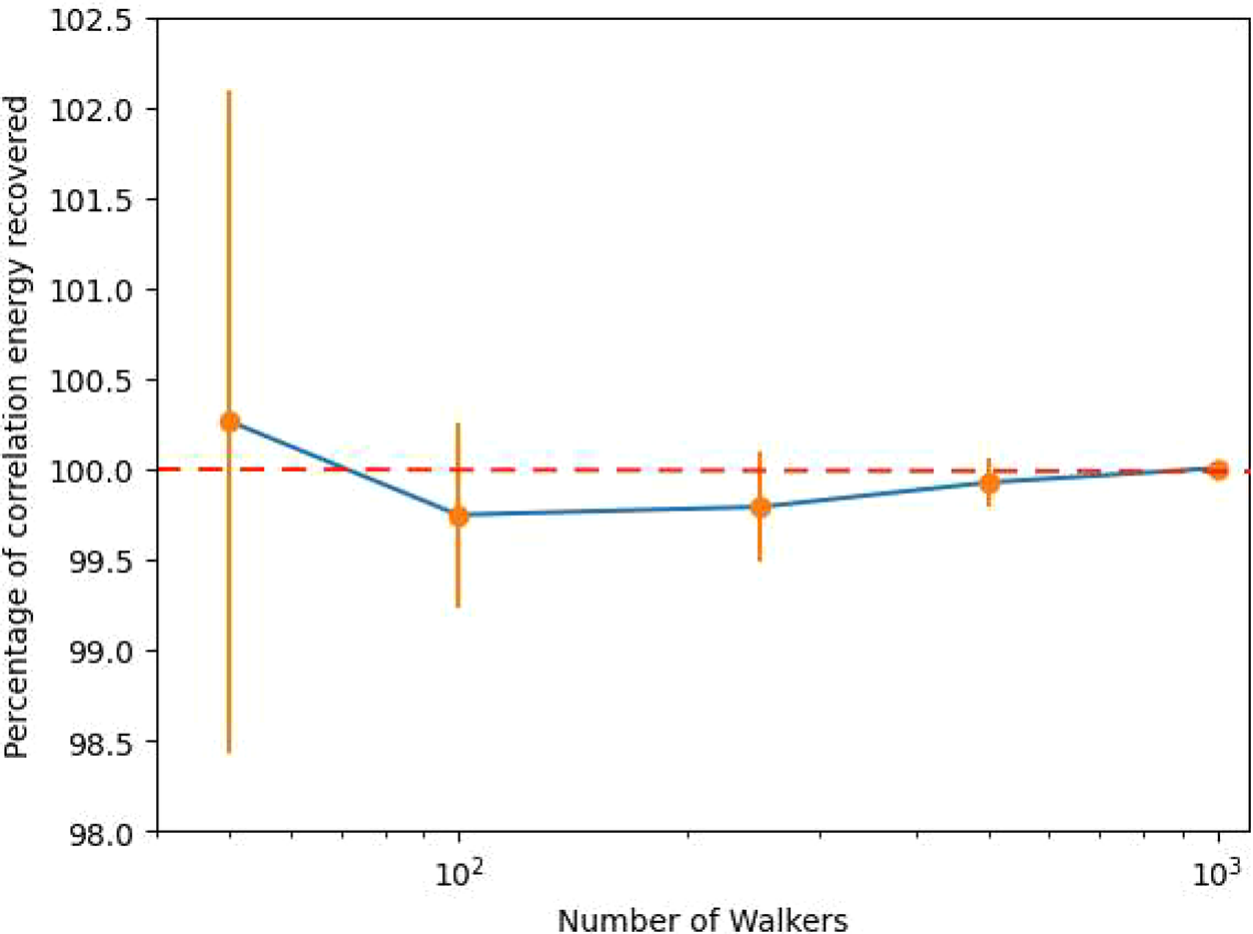
Relationship between the number of walkers and the percentage of correlation energy recovered relative to BE-FCI with error bars shown in H_8_.

According to the figure, one can deduce that the quality of the calculation is improved by increasing the number of walkers as the recovered correlation energy is closer to 100% and the stochastic error decreases. Both of these observations are similarly results of an increase in the granularity of the wave function with the number of walkers. It should be expected that this phenomenon is universal regardless of the chemical system, because the deviation in the values from the FCIQMC density matrix to the FCI density matrix should be zero in the large walker limit.

After performing the calculation in this simple test system, we set our sights on the scaling considerations of BE-i-FCIQMC, which is essential to understand if one wishes to perform BE-i-FCIQMC in realistic systems. The key question that needs to be answered is how the number of walkers scales with the Hilbert space if one wishes to maintain the same matching error and thus the overall quality of the calculation.

To answer this question, one might first hypothesize that to maintain the same matching error, the number of walkers should scale with the size of the Hilbert space of the fragments, meaning that the scaling is still exponential. Nevertheless, problems with respect to whether a reduced prefactor exists in the exponential scaling of walkers like FCIQMC and i-FCIQMC still arise. To answer them, seven larger systems are chosen so BE calculations can be performed on them with larger fragment sizes that contain 8, 12, or 16 atoms. Linear systems are chosen to exploit locality; while most of the atoms in the chains are hydrogen, some are replaced by either neon or fluorine atoms in a few systems, such that the Hilbert space sizes are identical for each of the fragments, which eases subsequent analysis; how the systems are constructed is outlined in Figure 5. BE2 and BE3 calculations are used in 8- and 12-atom system calculations, respectively. For BE3, as all of the atoms in the second coordination shells are considered, the fragment of a linear system now includes the central atom, two nearest neighbors, and two second-nearest neighbors, therefore containing five atoms. Similarly, one can argue that the type of embedding “BE4” that is used in calculating a 16-atom systems utilizes fragments that contain seven atoms. The coordinates of the systems are shown in the Supporting Information.

**Figure 5:**
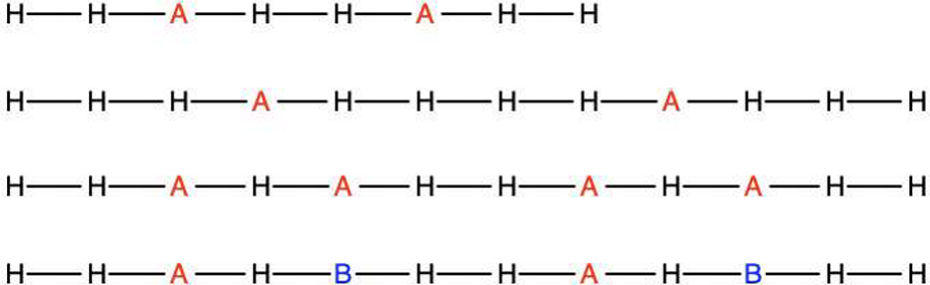
Way in which the systems are constructed to ensure that the fragment size is the same in H_6_A_2_, H_8_A_4_, H_10_A_2_, and H_8_A_2_B_2_, in which A and B are atoms other than hydrogen. The heteroatoms are colored red or blue.

After obtaining the relationship between the number of walkers and the minimum matching error that it can achieve, one can then plot to obtain an expression of the form log(*y*) = *a* + *b* × log(*x*), where *x* is the number of walkers and *y* is the matching error. This is naturally justified, as using an infinite number of walkers can, in theory, reduce the matching error to zero and work just like a deterministic FCI calculation. The Hilbert space sizes of individual fragments and the fitted parameters of the equations between the number of walkers and the matching error for the investigated systems are listed in Table 1. Excellent agreement can be seen from the fact that every *R*^2^ value in the plot for all of the eight systems investigated is ≥0.95, although it should be noted that these parameters are approximate due to the stochastic nature of BE-i-FCIQMC. Several conclusions can be reached using Table 1, which will be outlined below.

**Table 1:** Hilbert Space Sizes of an Individual Fragment and Fitted Parameters in the Nine Chemical Systems in Matching Error Estimations

chemical system	Hilbert space of a fragment	*a*	*b*	*R*^2^
H_8_	400	–1.526	–0.782	0.95
H_12_	6.35 × 10^4^	0.197	–0.840	0.99
H_16_	1.18 × 10^7^	–0.685	–0.464	0.99
H_6_F_2_	4.41 × 10^4^	–2.644	–0.440	0.96
H_6_Ne_2_	7.06 × 10^3^	–2.209	–0.708	0.99
H_8_Ne_4_	1.86 × 10^6^	–1.172	–0.549	0.96
H_10_F_2_	9.02 × 10^6^	–1.140	–0.549	0.97
H_8_Ne_2_F_2_	1.91 × 10^7^	–1.837	–0.557	0.98

According to the table, regardless of the sign of parameter *a*, the sign of parameter *b* is always negative. This means that the magnitude of matching error decreases monotonically with the number of walkers. This can be explained by an increase in the quality of the function and the decrease in the initiator error that is caused by the selected generation of particles, which is true regardless of the system.

In nonsubstituted systems such as H_8_, H_12_, and H_16_ in this work, the number of walkers required to reduce the matching error to 1.00 × 10^–4^, 1.00 × 10^–5^, and 1.00 × 10^–6^ can be calculated from the extrapolated parameters listed in Table 1. The relationship between the size of the Hilbert space and the amount of walkers required to achieve to the aforementioned matching error is plotted in Figure 6. According to the figure, one can see that the number of walkers required to converge to all three matching errors monotonically increases with the size of the Hilbert space of the fragment.

**Figure 6:**
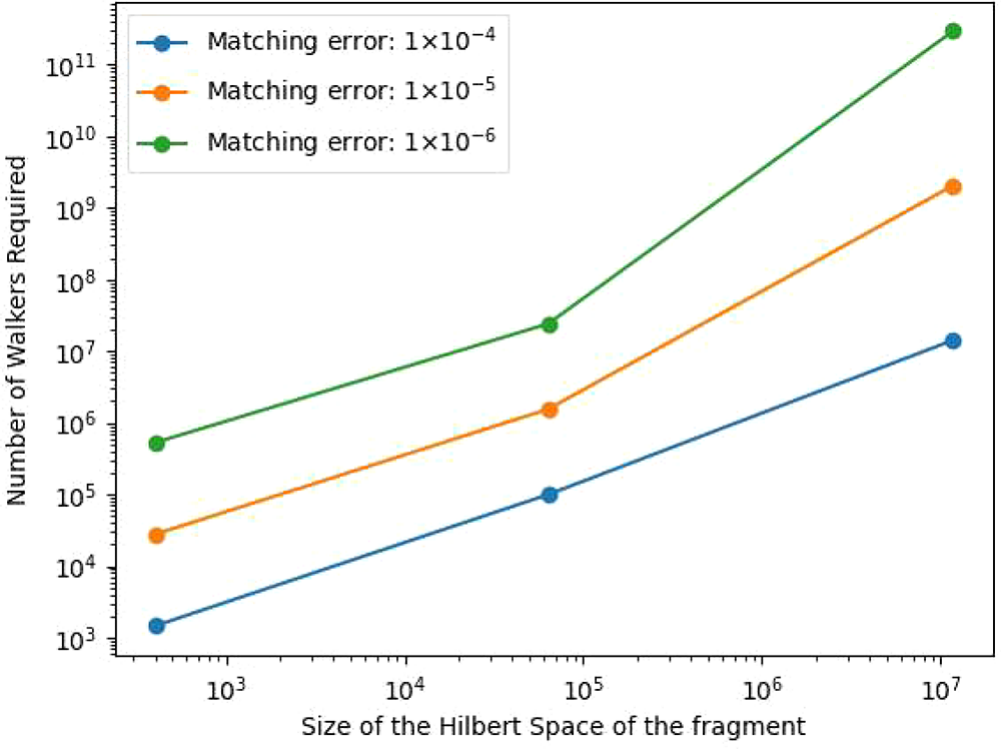
Relationship between the size of the Hilbert space of the fragment and the number of walkers required to reduce the matching error to 1.00 × 10^–4^, 1.00 × 10^–5^, and 1.00 × 10^–6^ in systems H_8_, H_12_, and H_16_.

Interestingly, one can observe that when the two types of heteroatoms, fluorine and neon, are included in the pure-hydrogen system, parameter *a* decreases significantly despite the sharp increase in the size of the fragment’s Hilbert space. The same phenomenon can also be observed in 12-atom systems. This means that lower matching errors can be achieved initially when compared to those of the pure-hydrogen chains, even though the corresponding Hilbert space sizes are larger for the fragments in these systems.

One might also plot the relationship between how the number of walkers scales with the Hilbert space when the target matching error is the same in the form of log(*y*) = *a* + *b* × log(*x*), where *x* is the size of the fragment’s Hilbert space and *y* is the number of walkers. The fitted parameters are listed in Table 2.

**Table 2:** Fitted Relationship between the Size of the Fragment’s Hilbert Space and the Number of Walkers to Reach a Matching Error

matching error	*a*	*b*	*R*^2^
1.00 × 10^–4^	1.433	0.533	0.46
5.00 × 10^–5^	2.382	0.570	0.47
1.00 × 10^–5^	2.789	0.586	0.47
5.00 × 10^–6^	3.739	0.623	0.44
1.00 × 10^–6^	4.146	0.639	0.43

Although the value of *R*^2^ suggested that the correlation is not so strong due to the dramatic difference among these systems, one can still see the positive correlation between the size of the Hilbert space and the number of walkers. In addition, one can see that both the intercept and the slope increase when the target matching error decreases, meaning that the advantage brought by a reduced prefactor decreases. This is not surprising, as the nature of the calculation would be closer to a deterministic FCI calculation.

Finally, to examine the ability of applying BE-i-FCIQMC in calculating realistic systems, two organic molecules, namely, benzene and cyclohexane, are chosen, and the level of theories are BE1-FCIQMC/6-31G* and BE1-i-FCIQMC/6-31G, respectively. As the sizes of the Hilbert space for the fragments are 1.40 × 10^15^ and 7.31 × 10^12^, they are too large for any deterministic FCI solvers to be implemented. BE1-i-FCIQMC calculations are done for benzene and cyclohexane using 5 × 10^5^ and 3 × 10^5^ walkers, respectively. The matching error thresholds for convergence are set to 1 × 10^–4^ for both of the compounds. The recovered correlation energies are then compared with deterministic BEn-CCSD (*n* = 1∼3), in which unrelaxed 1-PDMs are used due to their simplicity of generation, and all-electron CCSD and CCSD(T) using the same basis set. The results are shown in Figures 7 and 8, in which all of the correlation energy values are compared with the all-electron CCSD(T) correlation energy.

**Figure 7:**
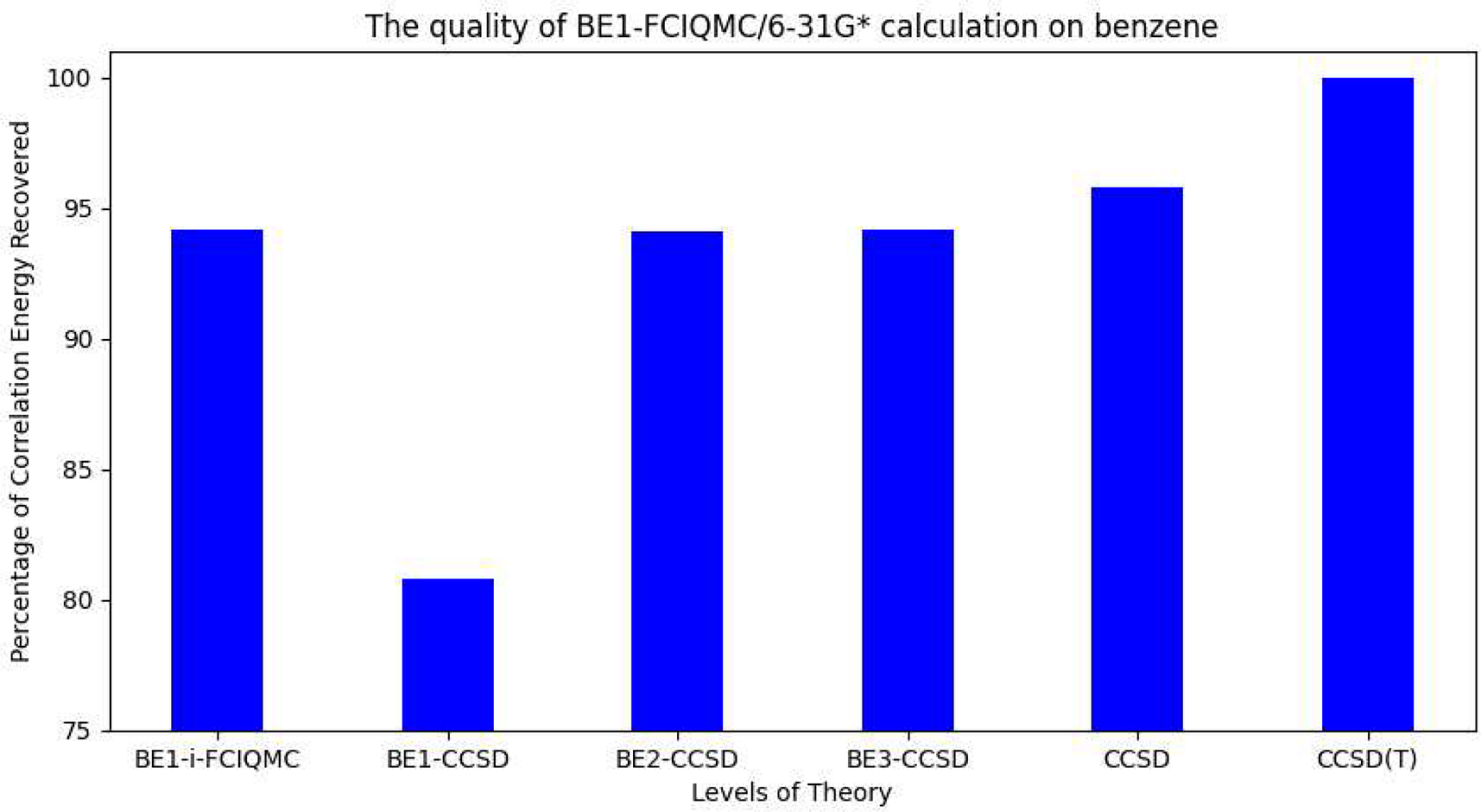
Relationship between the level of theory and the percentage of correlation energy recovered in benzene calculations. The all-electron CCSD(T)/6-31G* correlation energy is used as the reference value.

**Figure 8:**
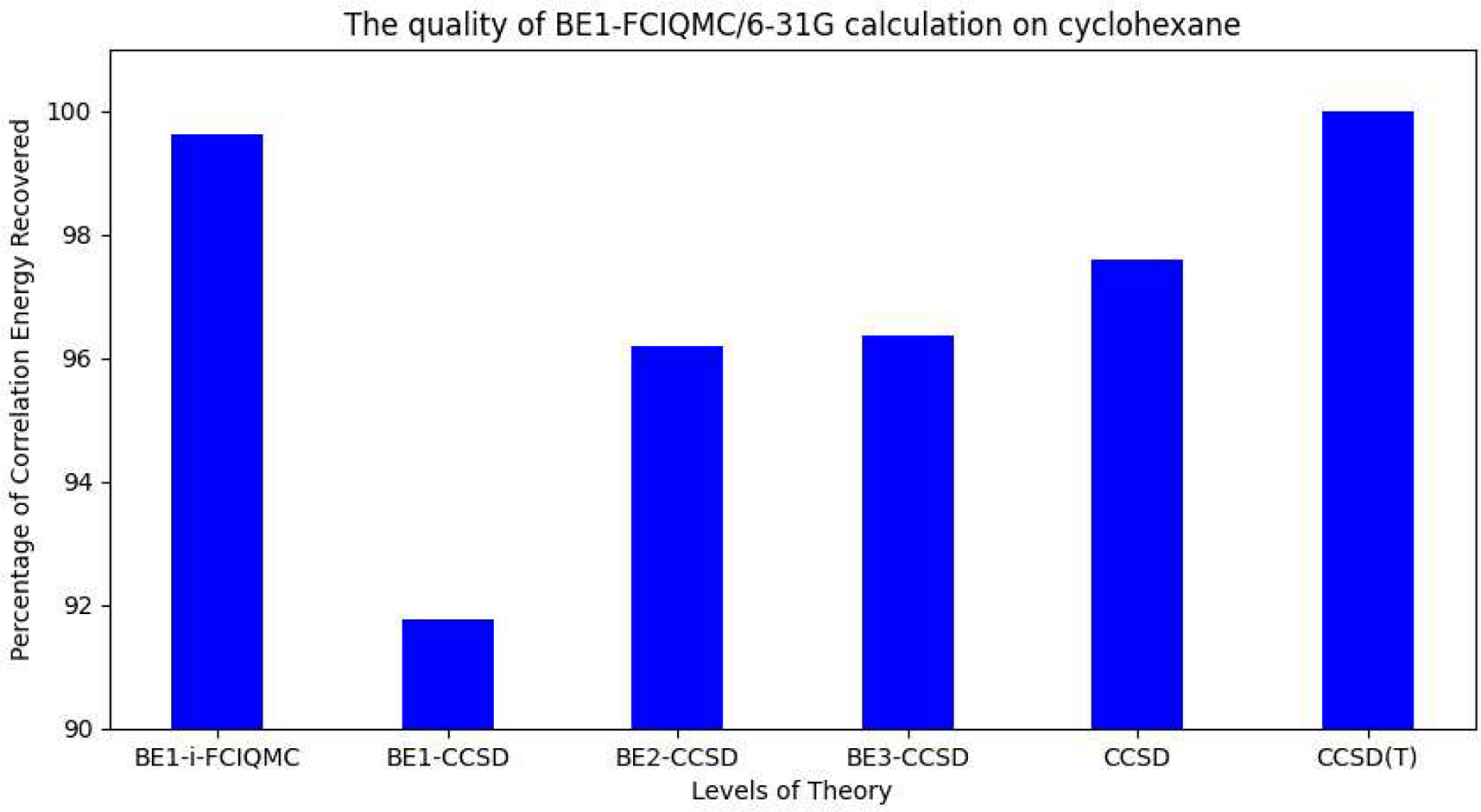
Relationship between the level of theory and the percentage of correlation energy recovered in cyclohexane calculations. The all-electron CCSD(T)/6-31G correlation energy is used as the reference value.

It can be shown from the graphs that even if the type of BE is 1, which is technically equivalent to density matrix embedding because there are no overlapping fragments, and each fragment contains only one carbon atom and its connected hydrogen atom(s), BE1-i-FCIQMC is already capable of recovering the correlation at the all-electron CCSD level in benzene and the all-electron CCSD(T) level in cyclohexane. BE1-CCSD performs poorly in both benzene and cyclohexane perhaps because of the incomplete sampling of the Hilbert spaces of the fragments. Although BE2-i-FCIQMC calculations are expected to perform better in these systems, the fragment Hilbert space sizes for benzene and cyclohexane would increase to 2.83 × 10^27^ and 9.40 × 10^27^, respectively, which are too large for the available computational resources to handle. Despite this, the aforementioned observations clearly demonstrate the potential of applying BE-i-FCIQMC in realistic molecules.

To summarize, we have successfully harnessed a stochastic solver to solve the BE equations and converge the BE calculations. To achieve this on a general system, one should first perform test calculations to determine the achievable matching error and then the threshold of the matching error to converge the calculations. We have first demonstrated that BE-i-FCIQMC calculations can perform as well as BE-FCI in the large walker limit, as both the quality of correlation energy recovered and the stochastic error monotonically decrease with an increase in the number of walkers. We then extended the calculation to a wider range of systems to show how the size of the fragment’s Hilbert space and the inclusion of other non-hydrogen atoms affect the number of walkers required to achieve the desired matching error. We finally implement BE-i-FCIQMC in realistic molecules, and it is inspiring to see that even BE1-i-FCIQMC can recover a similar amount of correlation energy or more correlation energy than BE3-CCSD, which utilizes much larger fragments. Future work should aim to use BE-i-FCIQMC to compute more realistic systems using more extensive computational resources to make calculations using BE2 or BE3-i-FCIQMC feasible, implement a stochastic solver in DMET, and use the stochastic analogue of coupled cluster theory, namely coupled cluster Monte Carlo (CCMC),^30^ as well as its variants^31−34^ in fragment embedding.

## Supporting Information Available

Method with which the 1- and 2-PDMs are sampled and coordinates of the chemical systems (PDF)
Transparent Peer Review report available (PDF)
