# Supplementary material for: Toward a Full Configurational Accuracy Calculation of an Arbitrary Molecule via Fragment Embedding and a Stochastic Solver
Source: J Phys Chem Lett. 2024 Apr 11;15(16):4249–55. doi: 10.1021/acs.jpclett.4c00634 (PMC11057031; doi:10.1021/acs.jpclett.4c00634)
Supplement: Supplementary file 1 — jz4c00634_si_001.pdf [file jz4c00634_si_001.pdf]

Supporting Information
Towards a Full Configurational Accuracy Calculation of an Arbitrary Molecule via Fragment Embedding and a Stochastic Solver

Yi Sun*

Department of Chemistry, Chicago Center for Theoretical Chemistry, James Franck Institute, and Institute for Biophysical Dynamics, The University of Chicago, Chicago, Illinois 60637, United States

E-mail: ys327@uchicago.edu

Simulation Details

Generating Integrals

An FCIDUMP file is generated for each i-FCIQMC calculation using PySCF to provide information regarding the electronic structure of the system when using stochastic quantum chemistry softwares such as NECI or HANDE. To understand how the FCIDUMP file of a 'normal' electronic structure calculation is different from solving a BE equation (11), it should be noted that although the two-electron terms are the same, the one-electron terms are affected by both the local effective potential and the global chemical potential term. Therefore, the corresponding FCIDUMP file of a BE equation (11) are generated for each fragment and it is subsequently parsed into the solver.

Sampling Particle Density Matrices (PDMs)

It should be noted that although one could always utilise the naive algorithm by evaluating the wavefunction of the system at the end of the calculation, 1 and 2-PDMs can be accumulated on the fly by realising that as 2-PDMs are expressed as

$$\Gamma_{pq,rs} = \sum_{\mathbf{ij}} C_{\mathbf{i}} C_{\mathbf{j}} \left\langle D_{\mathbf{i}} \left| a_p^{\dagger} a_q^{\dagger} a_s a_r \right| D_{\mathbf{j}} \right\rangle \tag{1}$$

in which $C_{\mathbf{i}}$ is the coefficient of an FCI determinant $\mathbf{i}$.[1] this value is non-zero if and only if $D_{\mathbf{j}}$ is connected by $D_{\mathbf{i}}$ by an $rs \rightarrow pq$ excitation. Therefore, to sample off-diagonal elements (sampling diagonal elements is trivial), a non-trivial contribution to an 2-PDM matrix element can be added if a successful spawning event occurs in $D_{\mathbf{i}}$ from $D_{\mathbf{j}}$, therefore the contribution could be expressed as

$$\frac{C_{\mathbf{i}} C_{\mathbf{j}}}{p_c \left(n_{\mathbf{j}}\right) (\mathbf{j} \mid \mathbf{i})} \tag{2}$$

in which $p_c \left(n_{\mathbf{j}}\right)$ is the normalised probability of spawning at least one child (of any weight) onto $D_{\mathbf{j}}$ from $D_{\mathbf{i}}$ during the current iteration. After the program starts to collect the PDMs, it will continue to do so for the next 200 iterations (1000 for the energy calculations in H_8, and 100 for the energy calculation in real molecules) before ending the calculation. The value of 1-PDM can then be obtained from 2-PDMs via tracing the diagonal matrix elements.

Running Calculations

For each i-FCIQMC calculation, the seed of the random number generator is randomly chosen from 1 to 10000. The shift is initially set to 0.1 by using the 'diagShift' option. The number of walkers is allowed to have non-integer calculations by enabling the option 'allrealcoeff'. With respect to the initiator approximation, the initiator threshold is set to be 3 so that any determinant with more than 3 walkers will be an initiator. A tau search for optimising it is also turned on using the conventional algorithm by enabling the option 'tau-search', and the initial value is set to 0.005.

Coordinates of the systems investigated

H_8

```
H 0 0 0

H 0 0 1

H 0 0 2

H 0 0 3

H 0 0 4

H 0 0 5

H 0 0 6

H 0 0 7
```

H_6F_2

```
H 0 0 0

H 0 0 1

F 0 0 2

H 0 0 3

H 0 0 4

F 0 0 5

H 0 0 6

H 0 0 7
```

H_6Ne_2

```
H 0 0 0

H 0 0 1

Ne 0 0 2

H 0 0 3

H 0 0 4
```

```
Ne 0 0 5
H 0 0 6
H 0 0 7
```

H_{12}

```
H 0 0 0
H 0 0 1
H 0 0 2
H 0 0 3
H 0 0 4
H 0 0 5
H 0 0 6
H 0 0 7
H 0 0 8
H 0 0 9
H 0 0 10
H 0 0 11
```

H_{16}

```
H 0 0 0
H 0 0 1
H 0 0 2
H 0 0 3
H 0 0 4
H 0 0 5
H 0 0 6
H 0 0 7
H 0 0 8
```

```
H 0 0 9
H 0 0 10
H 0 0 11
H 0 0 12
H 0 0 13
H 0 0 14
H 0 0 15
```

$H_{10}F_2$

```
H 0 0 0
H 0 0 1
H 0 0 2
F 0 0 3
H 0 0 4
H 0 0 5
H 0 0 6
H 0 0 7
F 0 0 8
H 0 0 9
H 0 0 10
H 0 0 11
```

$H_8F_2Ne_2$

```
H 0 0 0
H 0 0 1
F 0 0 2
H 0 0 3
Ne 0 0 4
```

H 0 0 5

H 0 0 6

F 0 0 7

H 0 0 8

Ne 0 0 9

H 0 0 10

H 0 0 11

C_6H_6

C	-7.55489	1.38567	0.00000
C	-6.50145	2.29989	-0.00000
C	-5.18299	1.84469	0.00000
C	-7.28988	0.01625	0.00000
C	-5.97142	-0.43895	0.00000
C	-4.91798	0.47527	0.00000
H	-8.58212	1.74033	-0.00000
H	-8.11063	-0.69603	0.00000
H	-3.89074	0.12062	0.00000
H	-5.76494	-1.50588	0.00000
H	-6.70792	3.36683	-0.00000
H	-4.36224	2.55697	0.00000

C_6H_{12}

C	-10.09400	0.99702	-0.09992
C	-8.75138	1.52478	0.40109
C	-7.65077	0.46959	0.32221
H	-8.85796	1.86010	1.43961
H	-8.46026	2.40221	-0.18815

C	-8.05924	-0.82671	1.01589
H	-6.73433	0.85933	0.78008
H	-7.41894	0.26190	-0.72933
C	-10.48520	-0.31526	0.57500
H	-10.04263	0.84529	-1.18467
H	-10.87245	1.74770	0.07982
C	-9.37765	-1.35875	0.46280
H	-11.40429	-0.70295	0.12082
H	-10.70520	-0.13034	1.63334
H	-9.24590	-1.64192	-0.58859
H	-9.66755	-2.26587	1.00500
H	-8.15943	-0.64984	2.09368
H	-7.27300	-1.57905	0.88652

References

(1) Overy, C.; Booth, G.H.; Blunt, N.S.; Shepherd, J.J.; Cleland, D. and Alavi, A. Unbiased reduced density matrices and electronic properties from full configuration interaction quantum Monte Carlo. *The Journal of chemical physics*, **2014** *141* (24).
