# Supplementary material for: Toward a Full Configurational Accuracy Calculation of an Arbitrary Molecule via Fragment Embedding and a Stochastic Solver
Source: J Phys Chem Lett. 2024 Apr 11;15(16):4249–55. doi: 10.1021/acs.jpclett.4c00634 (PMC11057031; doi:10.1021/acs.jpclett.4c00634)
Supplement: Supplementary file 2 — jz4c00634_si_002.pdf [file jz4c00634_si_002.pdf]

jz-2024-006348.R1

Name: Peer Review Information for "Towards a Full Configurational Accuracy Calculation of an Arbitrary Molecule via Fragment Embedding and a Stochastic Solver"

First Round of Reviewer Comments

Reviewer: 1

Comments to the Author

This interesting and well-written paper reports the implementation of combining bootstrap embedding (BE) with full configuration interaction quantum Monte Carlo (FCIQMC) as the high-level solver. Due to the stochastic noise of FCIQMC, the convergence of the BE equations is affected by the number of the walkers. The author investigates the effect of the number of walkers on the lowest convergence threshold that can be achieved for a range of one-dimensional systems. This demonstrates that the new approach can reach the same quality of convergence as BE with standard FCI.

The developments in this work will enable even larger systems to be modelled than when using standard FCI with embedding. The accuracy of BE-FCIQMC is demonstrated to be close to BE-FCI for a linear chain of 8 hydrogens by looking at the correlation energy. For the other systems only the convergence of BE-FCIQMC is investigated. It is not clear whether the approach will work well for realistic molecules as all the systems considered are linear chains although some of them contain neon and fluorine not just hydrogen.

The idea of using FCIQMC instead of FCI in embedding to treat larger systems is interesting and the technical implementation of BE with FCIQMC is impressive. The paper contributes an important investigation into whether FCIQMC, with achievable walker numbers, can work well with BE to calibrate the approach. I think it needs to present a more convincing case that this will enable FCI quality calculations on realistic systems, to make the work of broader interest. For example by

going beyond linear systems or applying BE-FCIQMC to accurately model linear systems with larger fragments that are too challenging for standard FCI.

The matching error shows how well the BE equations have converged and it is crucial to examine this when a Monte Carlo approach is used, as this paper does. It could be made clearer that a low matching error does not mean the BE approach is accurate as can be seen in Reference 19, Figure 4 where the matching error is 10^-6 but BE3-FCI does not describe a dissociation curve well. The accuracy will depend on the choice of embedding fragments and of the high-level solver.

It would also be useful to see the accuracy of the correlation energy compared with BE-FCI for some of the other systems in addition to H8. The Hilbert space of the fragments generally appears to be amenable to standard FCI, although the time cost will depend on the number of BE iterations required.

Could the author comment on the number of iterations needed to converge the BE equations to the chosen matching error for FCIQMC compared with FCI?

To provide more evidence that BE-FCIQMC has the potential to model realistic systems then a three-dimensional molecule could be considered and the challenges on moving from a chain of atoms discussed. For example, Reference 19 used BE-FCI for a potential curve of ethane and compared this with DMRG.

The illustration in Figure 1 looks like Figure 1 in reference 19 and the author should check if they need to cite the source of the diagram in the caption.

The supporting information on sampling particle density matrices in FCIQMC could cite reference 15. Perhaps it would also be useful to show how the 1-PDM is sampled or how it is calculated from the 2-PDM, as it is the 1-PDM that is used in the iterative BE equations.

The following are minor typos:

Figure 4 caption "percantage"

Page 10 "contain sever atoms"

page 12 "Flourine"

Reviewer: 2

Comments to the Author

The authors reported a numerical investigation of the bootstrap embedding (BE) method by employing Full Configuration Interaction Quantum Monte Carlo with the initiator approximation (i-FCIQMC) as a solver. This work is important for the quantum embedding community where there is a growing need for accurate yet computationally manageable solvers. The authors also discuss the scaling w.r.t. various computational parameters involved in a stochastic method to reduce the 'matching error'. This, I think is an important analysis for this solver. However, I think the presentation needs to be improved to appeal to the broader community. In this respect the following issues need to be taken care of:

a) It is necessary to show an example where one can't perform the BE-FCI calculations and, hence has to apply an approximate solver. In this context, a comparison of the FCIQMC solver with an existing affordable solver, for example, DMRG or CCSD will be useful.

b) The manuscript seems to be written hastily. There are quite a few broken sentences (for example, line 9 page 12), spelling errors (for example lines 27-28, page 10), very colloquial use of the words (line 17, page 7: runs instead of calculations) etc, which makes the manuscript less readable.

c) There are a few wrong references to figures, for example, in line 22, page 8 it should be Fig. 3.

Author's Response to Peer Review Comments:

Thank you for submitting your manuscript for publication in The Journal of Physical Chemistry Letters. It has been examined by expert reviewers who have concluded that the work is of potential interest to the readership of The Journal of Physical Chemistry Letters; however, it appears that a major revision, likely followed by further reviewer evaluation, will be needed prior to its further consideration for publication.

The authors thank the editor the providing the opportunity for further refining of the article. We believe that by addressing the concerns raised by the reviewers, the quality article should be suitable to be published in the Journal of Physical Chemistry Letters.

Please see the enclosed reviewers' reports for details regarding the requested changes and/or additions. I read carefully the reviews. All comments of both reviewers are important, but I would

wish to especially underline that i) having more relevant examples of applications, ii) providing comparisons with commonly-used solvers, are both likely needed to broaden the appeal of your work, which is important for our journal.

The authors have added more relevant examples in realistic molecules (benzene and cyclohexane), and have compared the BE-i-FCIQMC results with existing deterministic solvers. We believe that these changes will broaden the appeal of this article and will make it suitable for the publication in the Journal of Physical Chemistry Letters.

Besides, as mentioned by a referee, the Fig. 1 of the submitted contribution looks exactly the same as the one of van Voorhis' paper. It goes without saying that this issue should be fixed.

The authors have redrawn the Figure to fix this issue.

Please also make the following non-scientific changes:

1. Please include annotated version(s) of your revised publication file(s) with colored text or highlights indicating the revisions that you have made, and upload them as “Supporting Information for Review Only." Please also upload “clean” copies for publication. (No highlighting, annotations, or colored text permitted.)
2. Title: In both the main manuscript file and the Supporting Information, set the title in title case, with the first letter of each principal word capitalized.
3. Headers: Remove the section heading(s) throughout the body of the manuscript (you can leave Methods, Abstract, and TOC Graphic headings).
4. TOC Graphic: Provide a TOC image per journal guidelines (2 in x 2 in; on the same page as the abstract) with the heading “TOC Graphic” above the graphic. The graphic should be in the form of a structure, graph, drawing, photograph, or scheme—or a combination. Non-scientific cartoon-like images or caricatures are discouraged.

https://pubsapp.acs.org/paragonplus/submission/toc_abstract_graphics_guidelines.pdf 5. Graphics: Please consider using color in figure number 5.

6. Supporting Information Statement: A brief, nonsentence description of the actual contents of each supporting information file is required. This description should be labeled Supporting Information and should appear before the Acknowledgement and Reference sections. Examples of sufficient and insufficient descriptions are as follows: *Examples of sufficient descriptions:

“Supporting Information: 1H NMR spectra for all compounds” or “Additional experimental details, materials, and methods, including photographs of experimental setup”. *Examples of insufficient descriptions: “Supporting Information: Figures S1-S3” or “Additional figures as mentioned in the text”.

7. References: In both the main file and the supporting information, fix the style of all references to use JPCL formatting (check all references carefully). ***JPC Letters reference formatting requires that journal references should contain: () around numbers; author names; article title (titles entirely in title case or entirely in lower case); abbreviated journal title (italicized); year (bolded);

volume (italicized); and pages (first-last). Book references should contain author names; book title (in the same pattern); publisher; city; and year. Websites must include date of access.

8. Supporting Information: Please number SI pages in the following format: “S1, S2…”
9. Graphics: It seems that figure 1 has been reproduced or adapted from the publisher in Ref. 19.

Please provide the permission document from the publisher and upload it as a “for editors only” file.

Please also provide a reference citation in the caption in the following format: “Reproduced from [COMPLETE REFERENCE CITATION]. Copyright [YEAR] Publisher Name.”

These issues have been fixed by the authors.

Reviewer 1:

This interesting and well-written paper reports the implementation of combining bootstrap embedding (BE) with full configuration interaction quantum Monte Carlo (FCIQMC) as the highlevel solver. Due to the stochastic noise of FCIQMC, the convergence of the BE equations is affected by the number of the walkers. The author investigates the effect of the number of walkers on the lowest convergence threshold that can be achieved for a range of one-dimensional systems. This demonstrates that the new approach can reach the same quality of convergence as BE with standard FCI.

The authors thank the reviewers for believing the value of the paper. Indeed, this paper is about how FCIQMC could further empower BE so BE-FCI could be used in larger systems. We believe that, by adding two examples in calculating real-life molecules as well as improving the overall presentation, the work should be commensurate with the publication standard of the Journal of Physical Chemistry Letters.

The developments in this work will enable even larger systems to be modelled than when using standard FCI with embedding. The accuracy of BE-FCIQMC is demonstrated to be close to BE-FCI for a linear chain of 8 hydrogens by looking at the correlation energy. For the other systems only the convergence of BE-FCIQMC is investigated. It is not clear whether the approach will work well for realistic molecules as all the systems considered are linear chains although some of them contain neon and fluorine not just hydrogen.

The idea of using FCIQMC instead of FCI in embedding to treat larger systems is interesting and the technical implementation of BE with FCIQMC is impressive. The paper contributes an important investigation into whether FCIQMC, with achievable walker numbers, can work well with BE to calibrate the approach. I think it needs to present a more convincing case that this will enable FCI quality calculations on realistic systems, to make the work of broader interest. For example by going beyond linear systems or applying BE-FCIQMC to accurately model linear systems with larger fragments that are too challenging for standard FCI.

The authors have added examples in using BE-FCIQMC in calculating the energies of benzene and cyclohexane using a double-zeta valance set and have compared the results with several deterministic solvers. We believe that this proves the potential of using BE-FCIQMC in realistic systems.

The matching error shows how well the BE equations have converged and it is crucial to examine this when a Monte Carlo approach is used, as this paper does. It could be made clearer that a low matching error does not mean the BE approach is accurate as can be seen in Reference 19, Figure 4 where the matching error is 10^-6 but BE3-FCI does not describe a dissociation curve well. The accuracy will depend on the choice of embedding fragments and of the high-level solver.

The authors have added this remark in the paper as the response: 'The accuracy of the calculation will ultimately depend on the choice of the embedding fragments and the high-level solver.'

It would also be useful to see the accuracy of the correlation energy compared with BE-FCI for some of the other systems in addition to H8. The Hilbert space of the fragments generally appears

to be amenable to standard FCI, although the time cost will depend on the number of BE iterations required.

The authors have added this remark in the paper as the response: ‘It should be expected that this phenomenon is universal regardless of the chemical system, since the deviation in the values from the FCIQMC density matrix to the FCI density matrix should be zero in the large walker limit.’ Could the author comment on the number of iterations needed to converge the BE equations to the chosen matching error for FCIQMC compared with FCI?

The authors have added this remark in the paper as the response: ‘It is worth noticing that before the stochastic noise significantly affects the matching error, i.e., at a similar order of magnitude, the number of iterations needed to converge the BE-FCI and BE-i-FCIQMC to a designated matching error is similar.’

To provide more evidence that BE-FCIQMC has the potential to model realistic systems then a three-dimensional molecule could be considered and the challenges on moving from a chain of atoms discussed. For example, Reference 19 used BE-FCI for a potential curve of ethane and compared this with DMRG.

The authors have added examples in using BE-FCIQMC in calculating the energies of benzene and cyclohexane using a double-zeta valance set and have compared the results with several deterministic solvers. We believe that this proves the potential of using BE-FCIQMC in realistic systems.

The illustration in Figure 1 looks like Figure 1 in reference 19 and the author should check if they need to cite the source of the diagram in the caption.

The authors have redrawn the Figure.

The supporting information on sampling particle density matrices in FCIQMC could cite reference 15. Perhaps it would also be useful to show how the 1-PDM is sampled or how it is calculated from the 2-PDM, as it is the 1-PDM that is used in the iterative BE equations.

It has been mentioned in the remark: ‘The value of 1-PDM can then be obtained from 2-PDMs via tracing the diagonal matrix elements.’

The following are minor typos:

Figure 4 caption

"percantage" Page 10

"contain sever atoms" page

12 “Flourine"

The authors have also corrected the typos.

Reviewer 2:

The authors reported a numerical investigation of the bootstrap embedding (BE) method by employing Full Configuration Interaction Quantum Monte Carlo with the initiator approximation (iFCIQMC) as a solver. This work is important for the quantum embedding community where there is a growing need for accurate yet computationally manageable solvers. The authors also discuss the scaling w.r.t. various computational parameters involved in a stochastic method to reduce the 'matching error'. This, I think is an important analysis for this solver. However, I think the presentation needs to be improved to appeal to the broader community. In this respect the following issues need to be taken care of:

The authors thank the reviewer for believing in the value of the paper. We have improved the presentation of the paper by including calculations of real-life molecules (benzene and cyclohexane) using BE-i-FCIQMC and have improved the presentation overall. We believe that the paper is commensurate with the publication standards of the Journal of Physical Chemistry Letters after these changes.

a) It is necessary to show an example where one can't perform the BE-FCI calculations and, hence has to apply an approximate solver. In this context, a comparison of the FCIQMC solver with an existing affordable solver, for example, DMRG or CCSD will be useful.

The authors have included the examples (benzene and cyclohexane) where BE-FCI cannot be implemented (the fragment Hilbert space sizes are roughly 7*10^12 and 1*10^15) and have compared the results with BE1-CCSD, BE2-CCSD, BE3-CCSD, all-electron CCSD and all-electron CCSD(T). We believe that this is a key in proving the real-life applicability of the BE-i-FCIQMC solver.

b) The manuscript seems to be written hastily. There are quite a few broken sentences (for example, line 9 page 12), spelling errors (for example lines 27-28, page 10), very colloquial use of the words (line 17, page 7: runs instead of calculations) etc, which makes the manuscript less readable.

The authors thank the reviewer for mentioning this, and have corrected them accordingly.

c) There are a few wrong references to figures, for example, in line 22, page 8 it should be Fig. 3.

The authors thank the reviewer for mentioning this, and have corrected them accordingly.

jz-2024-006348.R2

Name: Peer Review Information for "Towards a Full Configurational Accuracy Calculation of an Arbitrary Molecule via Fragment Embedding and a Stochastic Solver"

Second Round of Reviewer Comments

Reviewer: 1

Comments to the Author

The new version of the paper and the response have addressed my previous questions. The paper now demonstrates that BE1-FCIQMC can recover more of the CCSD(T) correlation energy than bootstrap embedding with CCSD for realistic molecules (benzene and cyclohexane) when the embedding fragments are currently beyond standard FCI. I believe this increases the significance of the work and will be of general interest to physical chemists.

My minor comments are that it would be helpful to provide information on the number of walkers for the benzene and cyclohexane calculations, and also the matching error used. For BE-CCSD could the author add whether the unrelaxed 1-PDM or the response 1-PDM is employed?

Reviewer: 2

Comments to the Author

In this revision, the author has incorporated the results of calculations using two realistic systems: cyclohexane and benzene for the BE-i-FCIQMC method. The author also provides a comparison with one approximate solver, namely CCSD, for these examples. This certainly makes the paper more appealing to the broader community. However, I believe that some details about these calculations should be provided:

a) How many walkers were used for the cyclohexane and benzene examples, and what were the matching errors?

b) Why is the CCSD solver performing poorly for the BE1 calculations?

c) Naively, one might assume that BE2 calculations are perhaps more suitable for the benzene molecule due to its Π bonds, and the BE2-CCSD results appear to be quite reasonable. Would it be possible to show results for BE2-i-FCIQMC calculations for these systems? This would establish whether BE1 calculations are indeed sufficient.

I would recommend publishing this article after these issues are adequately addressed. Moreover, I suggest reconstructing some of the sentences for better readability.

Author's Response to Peer Review Comments:

Journal: The Journal of Physical Chemistry Letters
Manuscript ID: jz-2024-006348.R1
Original Submission Date: 27-Feb-2024
Title: "Towards a Full Configurational Accuracy Calculation of an Arbitrary Molecule via Fragment Embedding and a Stochastic Solver" Author(s): Sun, Yi

Dear Dr. Sun:

Thank you for submitting your manuscript for publication in The Journal of Physical Chemistry Letters. It has been examined by expert reviewers who have concluded that the work is of potential interest to the readership of The Journal of Physical Chemistry Letters.

Your revision was reviewed again and the referees have positively assessed the changes made. I note however that a further analysis of the results would help in showing the potential of this method. Please see the comments below.

The authors deeply thank the reviewers for providing positive reviews of the manuscript. We have made the changes mentioned by the reviewers to include more details in the applications of BE-iFCIQMC methods in realistic systems. We hope that the changes are sufficient for the paper to be published in The Journal of Physical Chemistry Letters.

We allow 30 days for revision, but we encourage you to submit within two weeks. Here at JPC Letters we try to expedite the processing of your manuscript, so your prompt response is greatly appreciated.

When submitting your revised manuscript through ACS Paragon Plus, you will be able to respond to the reviewers' and editorial comments in the text box provided or by attaching a file containing your detailed responses outlining the changes made and explaining your reasons for disagreeing with any suggestions you choose not to follow.

The Journal of Physical Chemistry Letters offers authors of Letter manuscripts an opportunity to participate in transparent peer review. Transparent peer review allows the reader to see the exchange between authors and reviewers. If an author chooses to participate in transparent peer review, the anonymous reviewers 'comments and author's response to the reviewers will be published as supporting information if the manuscript is accepted for publication. More information about transparent peer review can be found here https://pubs.acs.org/page/peer_reviews/transparent_peer_review.html or at a recently published editorial https://pubs.acs.org/doi/10.1021/acs.jpclett.1c03308.

During submission, you were given a choice to participate in transparent peer review. You responded as follows:

Yes, I will participate in transparent peer review.

If you opted to participate in transparent peer review, you can change your mind at any revision stage. If you have questions about transparent peer review not answered in our FAQs, please contact ACS Publications Support at support@services.acs.org or contact the editorial office.

To Revise Your Manuscript on the Web:

To revise your manuscript, log into ACS Paragon Plus with your ACS ID at http://acsparagonplus.acs.org/ and select "My Authoring Activity". There you will find your manuscript title listed under "Revisions Requested by Editorial Office." Your original files are available to you when you upload your revised manuscript. If you are replacing files, please remove the old version of the file from the manuscript before uploading the new file.

Format: Your revised manuscript must adhere to The Journal of Physical Chemistry Letters format. An author checklist is available at http://pubs.acs.org/paragonplus/submission/jpchax/jpclcd_checklist.pdf and The Journal of Physical Chemistry Letters author guidelines are available at https://publish.acs.org/publish/author_guidelines?coden=jpclcd.

Supporting Information: If the manuscript is accompanied by any supporting information for publication, a brief description of the supplementary material is required in the manuscript. The appropriate format is: Supporting Information. Brief statement in nonsentence format listing the contents of the material supplied as Supporting Information.

Funding Sources: Authors are required to report ALL funding sources and grant/award numbers relevant to this manuscript. Enter all sources of funding for ALL authors relevant to this manuscript in BOTH the Open Funder Registry tool in ACS Paragon Plus and in the manuscript to meet this requirement. See http://pubs.acs.org/page/4authors/funder_options.html for complete instructions.

ORCID: Authors submitting manuscript revisions are required to provide their own validated ORCID iDs before completing the submission, if an ORCID iD is not already associated with their ACS Paragon Plus user profiles. This iD may be provided during original manuscript submission or when submitting the manuscript revision. You can provide only your own ORCID iD, a unique researcher identifier. If your ORCID iD is not already validated and associated with your ACS Paragon Plus user profile, you may do so by following the ORCID-related links in the Email/Name section of your ACS Paragon Plus account. All authors are encouraged to register for and associate their own ORCID iDs with their ACS Paragon Plus profiles. The ORCID iD will be displayed in the published article for any author on a manuscript who has a validated ORCID iD associated with ACS Paragon Plus when the manuscript is accepted. Learn more at http://www.orcid.org.

We also require a graphic for the "Table of Contents" with all submissions. We ask that you include a graphic immediately after the Abstract under the header “TOC Graphic.” Instructions are available at https://publish.acs.org/publish/author_guidelines?coden=jpclcd.

ACS Publications uses Crossref Similarity Check Powered by iThenticate to detect instances of similarity in submitted manuscripts. In publishing only original research, ACS is committed to deterring plagiarism, including self-plagiarism. Your manuscript may be screened for similarity to published material.

Thank you for considering The Journal of Physical Chemistry Letters as a forum for the publication of your work.

With sincere regards,

Senior Editor
The Journal of Physical Chemistry Letters

Reviewer(s)' Comments to Author:

Reviewer: 1

Recommendation: This paper is publishable subject to minor revisions noted. Further review is not needed.

Comments:
The new version of the paper and the response have addressed my previous questions. The paper now demonstrates that BE1-FCIQMC can recover more of the CCSD(T) correlation energy than bootstrap embedding with CCSD for realistic molecules (benzene and cyclohexane) when the embedding fragments are currently beyond standard FCI. I believe this increases the significance of the work and will be of general interest to physical chemists.

The authors thank the reviewers the commanding the value and the general significance of the work.

My minor comments are that it would be helpful to provide information on the number of walkers for the benzene and cyclohexane calculations, and also the matching error used. For BE-CCSD could the author add whether the unrelaxed 1-PDM or the response 1-PDM is employed?

The details on number of walkers, the matching error, and whether the unrelaxed 1-PDM or the response 1-PDM is employed are mentioned in the remark: 'BE1-i-FCIQMC calculations are done for benzene and cyclohexane using 5×10^5 and 3×10^5 walkers, respectively. The matching error thresholds for convergence are set to 1×10^{-4} for both of the compounds, and unrelaxed 1-PDMs are used due to their simplicity of generation.'

Additional Questions:
Urgency: High

Significance: High

Novelty: High

Scholarly Presentation: Top 10%

Is the paper likely to interest a substantial number of physical chemists, not just specialists working in the authors' area of research?: Yes

Reviewer: 2

Recommendation: This paper is publishable subject to minor revisions noted. Further review is not needed.

Comments:

In this revision, the author has incorporated the results of calculations using two realistic systems: cyclohexane and benzene for the BE-i-FCIQMC method. The author also provides a comparison with one approximate solver, namely CCSD, for these examples. This certainly makes the paper more appealing to the broader community.

The authors thank the reviewers the commanding the value and the general significance of the work.

However, I believe that some details about these calculations should be provided:

a) How many walkers were used for the cyclohexane and benzene examples, and what were the matching errors?

This is mentioned in the remark: BE1-i-FCIQMC calculations are done for benzene and cyclohexane using 5×10^5 and 3×10^5 walkers, respectively. The matching error thresholds for convergence are set to $1{\times}10^{-4}$ for both of the compounds.

b) Why is the CCSD solver performing poorly for the BE1 calculations?

This is explained in the added sentence: 'The reason why BE1-CCSD performs poorly in both benzene and cyclohexane might due to the incomplete sampling of the Hilbert spaces of the fragments.'

c) Naively, one might assume that BE2 calculations are perhaps more suitable for the benzene molecule due to its Π bonds, and the BE2-CCSD results appear to be quite reasonable. Would it be possible to show results for BE2-i-FCIQMC calculations for these systems? This would establish whether BE1 calculations are indeed sufficient.

The authors understand that using BE2-i-FCIQMC would increase the accuracy of the calculations. Unfortunately, the Hilbert space sizes (2.83×10^{27} and 9.40×10^{27} for benzene and cyclohexane fragments in BE2-i-FCIQMC) are too large for the available computational resources to handle. This is mentioned in the added sentence: 'Although BE2-i-FCIQMC calculations are expected to perform better in these systems, the fragment Hilbert space sizes for benzene and cyclohexane would jump to 2.83×10^{27} and 9.40×10^{27} respectively, which are too large for the available computational resources to handle. Despite this, the aforementioned observations clearly demonstrate the potential of applying BE-i-FCIQMC in realistic molecules.'

I would recommend publishing this article after these issues are adequately addressed. Moreover, I suggest reconstructing some of the sentences for better readability.

Additional Questions:
Urgency: High

Significance: High

Novelty: High

Scholarly Presentation: High

Is the paper likely to interest a substantial number of physical chemists, not just specialists working in the authors' area of research?: Yes

----

FOR ASSISTANCE WITH YOUR MANUSCRIPT SUBMISSION PLEASE CONTACT:
ACS Publications Customer Services & Information (CSI)
Email: support@services.acs.org

Phone: 202-872-4357
Toll Free Phone: 800-227-9919 (USA/Canada only)

PLEASE NOTE: This email message, including any attachments, contains confidential information related to peer review and is intended solely for the personal use of the recipient(s) named above. No part of this communication or any related attachments may be shared with or disclosed to any third party or organization without the explicit prior written consent of the journal Editor and ACS. If the reader of this message is not the intended recipient or is not responsible for delivering it to the intended recipient, you have received this communication in error. Please notify the sender immediately by e-mail, and delete the original message.

As an author or reviewer for ACS Publications, we may send you communications about related journals, topics or products and services from the American Chemical Society. Please email us at pubs-comms-unsub@acs.org if you do not want to receive these. Note, you will still receive updates about your manuscripts, reviews, or future invitations to review.

Thank you.
